# Epistemic (in)justice, social identity and the Black Box problem in patient care

**DOI:** 10.1007/s11019-024-10194-y

**Published:** 2024-02-14

**Authors:** Muneerah Khan, Cornelius Ewuoso

**Affiliations:** Steve Biko Centre for Bioethics, University of Witwatersrand, Johannesburg, South Africa

**Keywords:** Black Box AI, Healthcare, Patient-centred care, Epistemic justice, Social identity

## Abstract

This manuscript draws on the moral norms arising from the nuanced accounts of epistemic (in)justice and social identity in relational autonomy to normatively assess and articulate the ethical problems associated with using AI *in patient care* in light of the Black Box problem. The article also describes how black-boxed AI may be used within the healthcare system. The manuscript highlights what needs to happen to align AI with the moral norms it draws on. Deeper thinking – from other backgrounds other than decolonial scholarship and relational autonomy – about the impact of AI on the human experience needs to be done to appreciate any other barriers that may exist. Future studies can take up this task.

## Introduction

Artificial Intelligence (AI) – described as the “simulation of human processes by machines” (Burns et al., 2023) – is increasingly being integrated into different industries. One of the earliest conceptualizations of AI was by the mathematician Alan Turing in the 1950s when he developed the “imitation game” to test if machines were capable of achieving human intelligence (Turing 1950). Initially, AI extended to *simple* systems that were limited to “if-then rules” (Kaul et al. 2020). Since the 1950s, AI has evolved to include complex algorithms such as Machine Learning and Deep Learning, creating a platform to optimize operations in a variety of industries (Kaul et al. 2020). These industries include financial services, aviation, insurance, telecommunications, and healthcare (Hastings, 2022). From recognizing abnormal transactional patterns for fraud detection in the finance industry (Regunath, 2021) to providing solutions to air traffic control during unfavourable conditions in the aviation industry (Saini, 2022), the uptake of AI has taken the world by storm. The impact of AI has been so prominent that it is said to be at the core of the Fourth Industrial Revolution.

Healthcare is one of the front-running industries likely to benefit from AI. The applications of AI in healthcare are numerous. Globally, AI is being more readily adopted in patient care to deliver personalized care, with a projected timeline of limited use by 2025 and extensive use by 2035 (Bohr and Memarzadeh 2020). However, the full adoption of AI to optimize and/or deliver tailored care to patients’ specific needs will likely be inhibited by the AI Black Box Problem. Broadly, “[a black box problem] occurs whenever the reasons why an AI decision-maker has arrived at its decision are not currently understandable to the patient or those involved in the patient’s care because *the system itself* is not understandable to either of these agents” (Wadden 2021). In other words, a Black Box is problematic – within the context of patient care – because of a *current* gap in *understanding* between the AI and the users of the AI, including the potential beneficiaries – like the patient – of the technology. This gap exists because *the system itself* is not understandable. This is not the same as one’s inability to understand a diagnosis (Wadden 2021). For example, symptomatic anaemia is understood universally much in the same manner, and an AI might suggest a blood transfusion as a suitable mode of treatment. However, what values of the patient did the AI consider in its output? Why did the AI conclude that a blood transfusion is warranted? This question would be relevant to certain cadres of patients, such as a Jehovah’s Witness.

Yet, the non-understandability of AI is not the only problem a Black Box creates. A Black Box problem also occurs because the system itself is *currently unexaminable* or *opaque*. The user cannot work their way back from the outputs to interrogate its justification for that output. What *degree of priorities* are placed on what values or variables? What *reasons* are provided for what prognosis?

The current inability to examine these AIs creates several gaps relevant to clinician-patient relationships. Notably, it creates a knowledge/information exchange gap. For example, what cannot be understood cannot be adequately shared between the machine, the user (clinician) and the benefactor (patient). Yet, sharing relevant material information about a patient’s clinical care is essential to developing trust between a clinician and a patient. It allows for transparency of clinical processes and promotes patient autonomy through the informed consent process.

The Black Box problem also creates a responsibility gap. The responsibility gap implies that a health professional cannot be held accountable, liable, and culpable for an (intentionally wrongful) action. To understand how, the reader should take note that control condition (that is, one is the agent of – or has sufficient control over – the action) and epistemic condition (that is, one is fully aware of the action) are core conditions for impugning responsibility (Neri et al. 2020). The black-box nature of AIs implies that clinicians can neither be in control nor know what variables are considered in an AI output. They cannot tell whether and what consideration an AI gives problematic variables like gender, race, orientation, etc., to prevent harmful consequences to patients (Afnan et al. 2021). Suppose a course of treatment is AI-generated. In that case, who should be responsible when something goes wrong if clinicians cannot be blameworthy for such actions? AIs lack the freedom and agency to be held (civilly) liable for their actions (Neri et al. 2020). Without an agent who can rightfully be liable for AI actions, wrongful actions can hardly be determined and redressed. Precisely, negligence frameworks require health professionals to compensate for actions they are responsible for (Sullivan and Schweikart 2019). Suppose health professionals and AI cannot be held accountable for AI-generated outputs. In that case, patients cannot be compensated for wrongs arising from such outputs.

The knowledge exchange and responsibility gaps undermine the requirement to foster a patient’s informed decision-making capacity and create an informed decision-making capacity gap in the process. A clinician cannot share information that they do not have/know, and a patient cannot understand what is not shared. Why is one course of treatment preferable? When understanding is absent, a patient cannot make informed health decisions.

The preceding knowledge exchange, responsibility and informed decision-making gaps raise tough questions regarding integrating AI into patient care. Should AI be used in patient care despite these gaps? Addressing this and similar questions is vital since they directly impact a clinician’s obligation to good clinical practice and a patient’s right to self-determination. Some studies have explored the implications of AI’s opaqueness for informed consent and have justified the need for alternative forms of consent to bridge the knowledge exchange, responsibility and informed decision-making gaps (Astromskė et al. 2021; Cohen 2020). Finally, a study has explained *the nature* of the Black Box problem by drawing on views about trust in African scholarship (Ewuoso, 2023). This manuscript draws on the moral norms arising from the nuanced accounts of epistemic (in)justice in decolonial scholarship and social identity in relational autonomy to interrogate whether and how AI ought to be used in patient care in light of the Black Box problem.

## Research design and method

This manuscript’s research design deserves to be clarified. This conceptual normative ethics paper adopts a philosophical analytic method to interrogate the question, “what do the moral norms arising from the nuanced accounts of epistemic (in)justice and social identity imply for *whether* AI ought to be used in light of the Black Box problem?” This method is not uncommon and has been defended by others as an equally relevant method for pushing the boundary of knowledge on any issue (Molina and Dds 2022; Vogelstein and Colbert 2020) or defending a thesis.

The manuscript has three parts. The first part is descriptive, describing the relevant ethical concepts in decolonial scholarship and relational autonomy and the moral norms that arise from them. The second part evaluates and applies these moral norms to interrogate the research question. In the final part, the manuscript addresses potential objections to the justifications provided in defence of the thesis itself.

The manuscript non-systematically used phrases like “medical AI and informed consent”, “Epistemic justice in decolonial scholarship”, “medical AI and epistemic justice”, etc., to retrieve relevant materials, including journal articles, dissertations, books, and web-based articles from databases like PubMed, PhilPapers, Google Scholar and the Wits Library. The search yielded over 100 articles that were carefully analyzed.

This project is being undertaken to address an identified research gap and contribute to advancing knowledge in this area. This is particularly important given the current relevance of AI and its increased uptake in healthcare. Notably, the view that writings of decolonial scholars and relational autonomists can be combined to yield valuable insights into how we use medical AI in patient care in light of the Black Box problem has not been explored previously, or at least to a significant degree. Reasonably, this should be of interest to academic scholars. There is also social value. Integrating AI into patient care in light of the Black Box problem is complex, with many social implications. Exploring ethical principles, which have received little attention, will allow this manuscript to provide insight into creating a more equitable, just, and sustainable system for integrating AI into society. Furthermore, this project will raise critical insight into the social components that influence the implementation of AI into healthcare beyond just empirical results. Through this contribution, this manuscript can help shape policies for AI use in patient care and influence decision-making processes to guide clinicians on whether and how to integrate medical AI into their practice.

Finally, there are different subsets of AI that may be used for patient care. They include Machine Learning and Deep Learning. Machine learning starts with a set of training data, which is fed into the AI technology. Once the data is available, the machine uses the available data to train itself to find patterns and make predictions following what it has learnt. The learning can occur either through supervised, unsupervised, or reinforcement models. Contrarily, Deep Learning algorithms are more advanced brain-logical complex forms of AI that have higher predictive power and the capacity to learn from un/structured large data sets, including continuous data, with *nearly* no human interference or oversight. Deep Learning’s, unlike Machine Learning, layers are hidden, which makes them black-boxed. The analysis below has implications *primarily* for the black-boxed Deep Learning.

## Epistemic (in)justice and decolonized scholars

Decolonial scholarship is vast, complex and generates many contestations concerning its moral imperatives, goals, and participants. For example, the targets of decolonization are contested. Notably, decolonization is sometimes conceptualized as an anti-western, anti-colonial quest to reclaim Africa’s agency and her intellectual, economic, infrastructural, political, and social freedom by shifting the continent to the post-colonial (or post-neocolonial) era. This is the way Ademola Fayemi and Macaulay Adeyelure (2016) describe decolonization, that is, as the “process of self-critical awareness of foreseeing, discovering and avoiding hegemonic institutionalization as well as mental colonization of concepts and disciplines in contemporary *African scholarship.”* Conceptualized this way, decolonization discourse becomes an exclusive reserve of African scholars, walling off the discourse from non-Africans.

Notice that the positionality from or the context in which this discussion is had also has implications for the moral imperative of decolonization. The moral imperative of decolonization is the norm regarding *what we ought to do to decolonize*. In African health decolonial literature, the moral imperative of decolonization entails grounding health and health research discourses that have significant implications for Africa in dominant knowledge systems on the continent. Furthermore, at the health and health research curriculum level, this imperative entails giving greater recognition to African knowledge systems in developing the continent’s health and health research curricula. This will also mean that African scholars are afforded primary roles in African health and health research. In the global health discourses, African knowledge systems will have equal status as other knowledge systems in the knowledge production that underlies key international health policies or feeds into global health discourses. At the health journals’ level, the contributions and perspectives of African scholars will have equal prominence in journal publications on global issues. This list is not exhaustive. Nonetheless, what we wish to outline here is that, foregrounded this way, decolonization requires recognizing, inviting and granting agency to Africa.

Decolonization is also sometimes described as a quest to reclaim colonized persons everywhere. In this regard, decolonized scholars, as such, are individuals everywhere who challenge any hegemony or silencing and adopt practices, theories and pedagogies that decentralize oppressive colonial power. For this reason, Oelofsen (2015) describes decolonization as “the change that colonized countries go through when they become politically independent from their former colonizers.” Nelson-Maldonado-Torres (2016) equally echoes a similar view in his description of colonization and decolonization as “key terms for movements that challenge the predominant racial, sexist, homo- and trans-phobic conservative, liberal, and neoliberal politics of today”. In this regard, decolonization is placed in the contest of the human quest for liberation from oppression and domination everywhere. On this account, the moral imperative of decolonization will include de-silencing voices, acknowledging, inviting, and enhancing the agencies of individuals in discourses and issues that have significant implications for them and global issues.

The goal of the preceding paragraphs is not to defend the right way to conceptualize decolonization conclusively. Instead, we highlight the relevant decolonization aspects for this project’s evaluative task. Nonetheless, it is still worth emphasizing – as demonstrated by different publications – that decolonization is commonly accepted as the effort to emancipate previously colonized nations, persons, and communities from the generational impacts of colonial thought, which continue to govern social, political, intellectual and economic structures (Bua and Sahi 2022; Nordling 2018; Von Bismarck 2012). In other words, although scholars conceptualize decolonization differently, a central point is that decolonization entails marginalization, silencing of certain groups, and the domino effect it has on building and progressing inclusive societies. Framed this way, epistemic justice in decolonial scholarship thus becomes a quest to decentralize knowledge production by giving equal recognition to various knowledge systems and knowledge producers on global issues.

Notably, this section draws on the thinking about epistemic (in)justice, primarily – although not exclusively – in the scholarship of decolonized scholars like Miranda Fricker, Frantz Fanon, Nelson Maldonado-Torres, Olufemi Taiwo and Linda Tuhiwai Smith. Fricker (2007) described this term in 2007 as, “a wrong done to someone specifically in their capacity as a knower”. Two primary forms of epistemic injustice exist i.e., testimonial and hermeneutical injustice (Fricker 2007). Testimonial injustice occurs owing to a credibility deficit influenced by an identity prejudice (Fricker 2007). For example, a female academic whose testament holds less value than her male counterpart owing to gender prejudice. On the other hand, hermeneutical injustice occurs because a person cannot express an important aspect of their social experience due to hermeneutical marginalization (Fricker 2007). For example, oppressed people often lack the necessary resources to make sense of their oppression, as was likely the case during the era of slavery.

The reader should observe that both the testimonial and hermeneutic injustices create a (i) disadvantage condition since it disadvantages the knower in relation to others, whose thoughts are given serious consideration in the knowledge production, and (ii) a prejudice condition, since such exclusion arises from biases against the speaker based on their say, race or gender. Since Miranda Fricker conceptualized the term, other conditions have also been added, (i) stakeholder condition (suppose the excluded knower has a stake in the outcome from which they are excluded because of biases against them), (ii) epistemic condition (the excluded knower does, in fact, possess the relevant knowledge), and (iii) social justice condition (that is, the bias against the knower is indeed connected to larger structural injustices like racism) (Byskov 2021). In light of this, the moral imperative of epistemic justice is *de-silencing* or *inclusion* that respects the agency of others, and is substantive and transformative. Precisely, epistemic justice in the decolonial narrative brings to the fore the necessity of including the voices and knowledge systems that were previously dismissed based on identity prejudices and marginalization, enabling credibility that previously did not exist, not for lack of knowledge, but for lack of affirmative presence, and understanding how silencing or domination occurs.

Silencing is complex and occurs in various ways not limited to mythologizing Western superiority. The preceding reaffirms the view that the West is the only legitimate and civilized source of knowledge (Smith 1999). Silencing could also occur through cognitive injustice, which is the systematic exclusion of the values and beliefs of those dominated in the knowledge production that feeds into many practices. For example, to mitigate the effect, colonial education was used as a tool to create “indigenous elites”, on the presumption that these elites would align their interests with colonizers (Smith 1999). This effectively resulted in a dismissal of knowledge and culture that represented where they came from.

To undo the harm of silencing and foster a more transformative inclusion, all people, irrespective of who they are or where they come from, ought to be equally included in the development, progression, and distribution of knowledge, especially if they have a stake or may be significantly impacted by the outcomes that are informed by such knowledge production. This means additional focus needs to be placed on marginalized groups, who have thus far not received the necessary credibility for their contribution to knowledge production. They often lack the resources to receive knowledge that may be valuable to them to make sense of their social experiences.

Finally, it calls for fair distribution of power and resources. To understand how the reader will recall that epistemic injustice is often connected to the larger structural injustices (or social justice condition). Social disadvantage is a generational trauma and may influence identity prejudice. As Táíwò (2020) stated in an essay, the goal (of epistemic justice) is to build (new) rooms where people can sit together rather than limit themselves to navigating around rooms that history has made. Suppose power is what it takes to be in a room where influence occurs. In that case, power should be distributed to all, not merely by representation of marginalized groups in the room but by including the marginalized in developing standards for re/distributing power.

### Social identity and relational autonomists

This section outlines the moral norms that can arise from the thinking about social identity grounded in the scholarships of relational autonomists. Autonomy has long been an integral part of ethics and philosophy, with depictions tracing back to ancient Greek Medicine and later to the philosopher Immanuel Kant. In 1979, it reached its pinnacle when Beauchamp and Childress (1979) added “Respect for autonomy” as one of the four principles in their book *Principles of Biomedical Ethics*, which became a foundation upon which modern ethics of medicine would be built. In their book, autonomy is conceptualized as the right to self-determination that often necessitates moral rules like the requirement of informed consent and respect for one’s privacy. Varelius (2006) echoes a similar view in their description of autonomy as self-governance and further describes personal autonomy as “self-rule that is free from both controlling interference by others and from limitations, such as inadequate understanding, that prevent meaningful choice”. This is in keeping with the traditional concept of autonomy, which is aligned with Western Individualism.

The Western-individual model of autonomy was progressed to overcome paternalism and the undue influence that authoritative figures could place on decision-making. However, it problematically ignored “values [like] mutual responsibility, cooperation and care towards others” (Dove et al. 2017). It assumes an impoverished view of humans as isolated beings, unbounded by any roots.

In recent years, however, this Western-individual model of autonomy has been challenged by feminists, communitarians, and identity politics theorists (Christman 2004). For example, Nedelskyt (1989), contended that people’s social and political relations impact how their predispositions, interests, and autonomy develop. These ultimately affect how people process information and use it to make crucial decisions – such as those that can be expected in healthcare and research. Through such discussions, a new concept of autonomy has emerged, namely relational autonomy. Prominent scholars contributing to this relational autonomy concept are Jennifer Nedelsky, Catriona Mackenzie, Natalie Stoljar, John Christman and Marina Oshana.

Catriona Mackenzie and Natalie Stoljar (2000) have described relational autonomy as an umbrella term that encompasses a similar perspective based on the premise that people are socially embedded and that a person’s identity manifests through their relationships and shared social factors. In other words, people understand who they are based on contextual factors such as religious affiliations, family values/connections, and community normative practices. As Baier (1985) claims, people are actually “second persons”, who succeed each other, and their personalities are uncovered through their relations with others.

Whereas autonomy has affirmatively been a process that discourages any external influence in fear of simulating paternalism, which modern medicine has moved away from, relational autonomy moves away from this stringency to a model that encourages engagement and shared decision-making. It suggests that involving others in important decisions does not conflict with being autonomous. Rather, this affirms the social nature of the individual (Walter and Ross 2014).

The preceding gives rise to the concept of social identity. Notably, social identity is the sense of self that develops through belonging to a group (Mcleod 2023). It (social identity) is a recurring theme among various relational autonomists. Equally, it is a key influence on how people make decisions.

However, the influence of the social component in relationality is not consistent among relational autonomists, and two broad categories of philosophical thinking have been noted. These are “causally relational” and “constitutively relational” accounts (Baumann 2008). *Causally relational accounts* suggest that social conditions act as background conditions for realizing autonomy without changing the definition of autonomy itself. In contrast, *constitutively relational accounts* believe that the connection between a person’s autonomy and social environment is more fundamentally entwined with “social” forming part of the definition (Baumann 2008). The overarching theme, however, among relational autonomists is that people do not exist in isolation and are a product of their social environment. A key difference lies in how much weight the social aspect plays in defining autonomy.

Suppose social identity in the works of relational autonomists implies that an individual’s web of connections and relations plays a crucial role in self-conception and understanding. In that case, one ought to honour individuals’ relations. Individuals are not just *isolated, independent* entities, but exist in relations and inter-relations. These relations define and redefine one’s identity as *social.* Notably, social identity is that certain individuals understand their place in the world as related and interrelated (Kajee 2010). Fragmenting the individual from their social reality, relations or embeddedness gives rise to a crisis of identity and is dislocating (or silencing). Social identity is a key part of their meaning-making. It also implies that the weight of decisions should be shared with relevant people who can assist in making life-altering decisions.

Furthermore, understanding people as a product of their social self, means that people are not simply accountable to themselves but to those around them who are affected by their decision. For example, a positive result from a genetic test done at an individual level – from the relational autonomy perspective – is relevant at a familial level as a risk factor to other family members. Dove and colleagues (2017) describe a familial approach that draws on relational concepts of autonomy and “emphasizes relational values, such as mutual responsibility (as a manifestation of reciprocity), and interdependence,” to highlight circumstances in which it may be appropriate to share sensitive information with others, particularly family members. This is because individuals tend to have “altruistic tendencies” and generally favour the familial approach (Dheensa et al. 2016), highlighting a sense of responsibility towards more than just themselves.

## Epistemic (in)justice and social identity: a new moral norm

At first glance, epistemic (in)justice and social identity appear to be distinct moral concepts or, at most, loosely related. However, the moral norms that underlie these concepts are closely linked, and in this section, we will highlight the overreaching moral norm that arise from the combination of these concepts.

Social identity represents a fixed set of values, rules, and commitments an individual adopts based on their origins. As argued by relational autonomists, it constitutes or influences one’s development of autonomy and ability to make choices regarding themselves. The choices made, in itself, are an expression of knowledge. The choices made by individuals represent the history of a person, cultural values, and community norms. These choices, at times, may not align with popular or contemporary views associated with the West but are equally valuable sources of knowledge. Conceptualizing, incorporating and distributing this knowledge will be essential in ensuring inclusivity and promoting epistemic justice.

Knowledge is not only an individual prerogative but one that can be progressed through families, communities and their leaders thereof. Inclusivity encompasses the acceptance that individuals may choose to source their knowledge from their close network as a trusted source, and use it to progress their decisions. The credibility of this knowledge should not simply be dismissed based on identity prejudices. Perhaps, a community leader who influences a decision is not merely patriarchal and paternalistic but one experienced enough to add value to the decision. Perhaps, an individual respects that leader, and does not feel pressured but reassured by their input.

Individuals and communities ought to receive a fair distribution of power and resources so that the social narrative changes to one where people are empowered with equal knowledge to develop their autonomy further. Access has long-defined social circumstances, and the lack thereof has led to a poor understanding of certain social experiences. In the face of contemporary medicine, individual autonomy increasingly poses the risk of isolation and trepidation (Ho 2008), making the need to consider social context more critical than ever.

The moral norms underlying epistemic (in)justice and social identity in the work of relational autonomists call for inclusivity of knowledge and sources of knowledge, empowerment of autonomy through equal resources, and respect for people and their values. In the next section, we will draw on this moral norm to interrogate, (i) the permissibility of integrating medical Black Box AI, particularly in patient care, (ii) how AI, particularly the more advanced ones like Deep Learning algorithms, ought to be integrated in the healthcare system, suppose this is permissible from the perspectives we draw on, and (iii) outline concrete actions which ought to be taken; what changes need to occur to align AI broadly with the norm we articulate in this section.

### Medical Black Box AI use for patient care: normative assessment

Three things regarding data are worth highlighting when normatively considering the use of AI in clinical care in light of the Black Box problem – (i) the origin of the training data put into the machine, (ii) how the machine processes the data available, and (iii) whether the AI output provides sufficient relevant information for an informed decision to be made. Although we recognize various other important uses of medical Black Box AI, in this section, we specifically demonstrate how the moral norm we articulate in the previous section yields the conclusion that medical Black Box AI use for *patient care* is problematic for the following reasons, (i) given that un/recognized prejudices may structure source data or operational environment, (ii) medical Black Box AI internal operations are opaque, and (iii) this opaqueness undermines the moral norm that we described previously.

Sources and inclusivity of knowledge are important considerations in the origin of training data. The gap in knowledge that the AI creates undermines this. Notably, suppose the AI learns based on the knowledge it is provided with. In that case, it becomes imperative that some level of transparency is exhibited by data generators and creators of the AI technology on what sources of knowledge are being used to collect and group data. What knowledge production is employed to develop standards used to group and collect data?

Furthermore, suppose the machine learns from the ongoing data it is fed or from the environment (as is the case with Deep Learning), which would likely occur through repeated use in various clinical subsets. In that case, the patients in those clinical settings may become one source of data. Health disparities would directly impact the collection of data in this manner. Health disparities refer to “differences between groups in health insurance coverage, affordability, access to and use of care, and quality of care” (Ndugga and Artiga 2021). This would include a lack of access to advanced technology to complement their healthcare. As such, there would be an underrepresentation of these groups of people in the data set, and health nuances specific to these people would be omitted. This could affect the accuracy of the output, given that a machine cannot consider unique factors it is not exposed to.

Some may argue that these might be the exception. However, many small exceptions may converge into big omissions. Advanced AI can learn from the environment or field operation in addition to the training data. Even this is problematic since certain prejudices remain in the healthcare context that continue to structure healthcare delivery in ways that disadvantage certain groups or individuals. Specifically, studies show a correlation between ethnicity, health and wealth in healthcare delivery (Hague 2019). The more advanced AI may learn this pattern and predict lower health outcomes for already disadvantaged individuals like the poor, thus causing limited medical resources to be withheld from them. Given the knowledge gap created by medical AI Black Box, it would be (nearly) impossible to detect when a Deep Learning algorithm has behaved unethically this way to correct it.

Secondly, even if the limitations in sources of data had to be overcome with conscientious input of training data that was fair and equitable, there lies the problem in how the Black Box processes use data to produce an output or rank outputs, thus, undermining control requirement for accepting responsibility for AI. Suppose there is no discernible way to know what factors were considered in assimilating the outcome. Equally, suppose epistemic (in)justice and social identity as a subsidiary of relational autonomy concur that people and their values should be respected. In that case, (to enable respect) a person’s values and personal nuances need to be considered in decisions made about them, or in this instance, in outputs that may influence their healthcare decisions. The knowledge-gap undermines the possibility of gaining this knowledge. At best, it can be assumed that if the output is accurate and in keeping with the patient’s values, then the machine might have considered all contributing factors. However, “assumed” and “might” are non-definitive terms. Such an output could also be purely co-incidental and may not have considered factors important to the patient at all. Hidden processes are counterintuitive to the concept of respect – of people, their values, their ideals – and Black Box AI represents just that through its opaque nature.

Another problem that the Black Box presents is the amplification of bias over an extended period. This may result from the intentional or unintentional introduction of biased training data. Suppose a machine that has not been calibrated recently is used to measure the vital signs of patients, and faulty data is inputed into the device over some time, the machine may incorrectly define a new normal based on the data it was fed. Or suppose stereotypical assumptions about groups of patients (for example, patients > 60 years have decreased renal function) are used to create the dataset that will be used to train the machine. The machine will eventually produce all outputs based on these stereotypical assumptions. This could radically alter the algorithm and skew all future outputs to favour this biased data. The Black Box hinders control over the exponentiation of the bias since the system used to process the information is not controlled by the creators nor understood. Therefore, the output produced may be evidently biased or may potentiate existing real-world biases, which adversely affects the drive for inclusivity. Quality control of data sets may assist in minimizing the potential for bias. However, the threat cannot be entirely eradicated without the ability to quality control the processes as with Black Box AI.

Ultimately, medical AI is only as good as its training data and the context it functions or learns from. Even if full control of data is achieved, what the machine does with data will still be a mystery – one where the opaqueness neither promises inclusivity of knowledge nor respect for people and their values. Nevertheless, even if it did have a higher awareness of such concepts, the Black Box conceptually makes it impossible to know or understand what more needs to be done to ensure that individuals’ social relations form the basis of AI predictions or accept responsibility for AI outputs.

Thirdly, there is a margin for concern in what should be done with the AI output once it has been processed. In a *patient healthcare* setting, that output could range from organizing patient files to suggesting a potential treatment or prognosis. However, in the *healthcare delivery* context, it could range from triaging emergency response or care services to planning clinical rotations. However, when directly linked to patient care, patients are usually expected to make an informed healthcare decision based on the output produced by the Black Box AI. This is problematic as the word “informed” suggests that sufficient information is required to be shared with the patient regarding how a certain outcome or decision was reached, so any decision taken based on it would be with good rationale. Given the informed consent gap that AI creates, the clinician using the AI would be unable to relate such information. Even if the clinician had to seek clarification from the creator, it would surmount to little as the Black Box can neither be opened nor understood. This would fail to meet the minimum requirement for informed consent, and any decision taken based on this output would merely be consent. Patients’ autonomy ought to be empowered through the distribution of equal resources and/or information. Forcing patients to make decisions about their health based on sub-minimal information goes against this principle.

Additionally, there is the social self that exists within a network of connections. As such, patient care is not an individual prerogative, and any decision made may affect several closely linked relationships. Can AI conceptualize the complexity of social self in processing information to suitably suggest patient care interventions that are beneficial on an individual level and holistically in keeping with the patient’s family dynamic? Given the nature of the Black Box, is it even possible to know? Beyond that, suppose that the patient in question is unconscious or incapacitated, and a proxy is appointed to decide on the patient’s behalf. The proxy would face the dilemma of deciding for someone else based on essentially opaque reasoning. For example, the AI has determined that the patient should be taken off end-of-life care. Therefore, the family should agree to it. A further explanation would be warranted. However, it would be limited to the interpretation of how the clinician “thinks” the AI came to that suggestion.

Furthermore, introducing Black Box AI into community healthcare programs in socially disadvantaged and rural communities may have added perplexities. Suppose a community with little to no contextual background knowledge of AI technology is now informed that a machine will be processing their information and assisting with their healthcare. They will lack the ability to make sense of this new social and healthcare experience fully. Two things may result. They could either overestimate its potential and be in blind favour of the AI-suggested output. Or they could implicitly refuse the added dynamic to their care based on mistrust of technology they are not familiar with, further contributing to a lack of representation within the training data set or healthcare context that continues to be structured by prejudices and histories of imperialism. It would also raise the question on whether the AI was built to consider minorities, and given the nature of the Black Box, it would be difficult to tell. That said, the existing output may already be biased given that the training data and the environment would be reflective of socially advantaged standpoints or prejudices where it existed before its introduction into underprivileged communities.

Given the moral norms arising from epistemic (in)justice and social identity, the above depicts that using AI *in patient care* in light of the Black Box problem can be deeply problematic, at least from these points of view.

### What needs to happen

A critic may be correct to observe here that this manuscript will not have the impact it intends in the AI community since it has not, at least to a significant degree, recommended concrete steps for aligning AI broadly with cherished values in the scholarship on decolonial and relational autonomy literature (or the concrete suggestions for addressing the gaps medical Black Box AI creates). This section interrogates this question. Specifically, it reflects on what concretely needs to happen to address the Black Box AI problem broadly; at the level of the knowledge production that informs how standards are developed for structuring data collection, gathering or training for AI; at the level of AI developers/creators since they may also have un/recognized prejudices that can feed into their creation; at the level of AI users like health professionals that implement AI predictions or the hospital administration that recommend AI use; at the level of the potential beneficiaries of AI for whom AI is used to optimize care; and at the level of policymakers and institutions who have the responsibility of safeguarding human welfare.

What needs to happen to address the gaps that medical Black Box AI creates and ensure that AI use promotes the moral norms this project draws on is that Black Box AI must become interpretable. The preceding would help, but we do not think this would decisively solve the Black Box problem, as we explain below. Moreover, research on more advanced AI interpretability has not yielded material success. In fact, as Tilman Räuker and colleagues (2022) have confirmed, current research on AI interpretability has been mostly unproductive. Under this assumption, what more could be done? What concretely needs to change to foster cherished human values?

First, inclusivity of knowledge production requires that both skilled professionals and the common man be included in the development of standards so that the value of human experience is not overshadowed by scientific superiority, given that both are valuable sources of knowledge. The preceding point does not imply that scientific evidence ought to be rejected. Notably, the preceding progresses the idea, that combining the two – scientific evidence and knowledge from the common man – could produce a standard of knowledge and patient care that is evidence-based, while still importantly respecting people and their values It also allows for the gap to be bridged between those who fall on either extreme, as their experience of the same technology will be significantly different. Furthermore, in order to empower groups of people to appreciate the significance of AI technology and to affiliate it as a social experience that may soon be available to them, it is imperative that they be included in setting the standards upon which training data should be developed. This can be done through advisory boards open to all people irrespective of their level of formal education and must be reflective of different sub-sets of the population likely to benefit from the technology.

At the level of AI developers/creators, there needs to be a diversity of the team working on developing AI. Movements and proclamations by companies globally are already driving towards employment diversity. However, its impact has been limited thus far. Human resources need to develop policies that seek out marginalized groups, and more educational resources need to be made available to these groups to develop them for skilled roles such as AI development. Women, people of colour, and transgender communities, amongst others, are an important part of the population, and including them on the team of AI developers will be more impactful than simply asking their opinion.

Critical thinking needs to occur at the level of the users, such as clinicians who implement and recommend AI outputs to patients. As powerful and accurate as AI in patient care might be, there is still room for error and bias. Clinicians need to be hypervigilant in its use and to trust only what they can rationalize through their experience while considering the human patient that they are managing. That said, an AI may not know that the patient is a Jehovah’s Witness, but a clinician would know that and should be able to practically deduce that something like a blood transfusion would not be feasible for said patient. Although this suggestion is important, we do not think it is sufficient. Notably, this suggestion is only relevant when a human is in the loop. As we explain in a subsequent section, the context is changing, and the reality is that some advanced AI can now function autonomously.

The clinician also needs to be trained to recognize biased and inaccurate output, and a concrete feedback loop needs to exist between the creators and users of AI so that previous shortfalls may enrich new knowledge production. The fear remains that new clinicians entering the healthcare system in the era of AI will have increased confidence in AI and concurrently lack the experience to differentiate otherwise.

Patients, as the AI’s benefactors in patient care, need to also play an active role in the feedback cycle, with a platform to express their satisfaction and grievances on the user experience. Furthermore, a patient may be the most suitable candidate in the AI life cycle to recognize prejudice especially if that prejudice is directed at them.

Balancing the human factors to use Black Box AI in a morally permissible way is a task that requires ongoing vigorous work and evaluation. Nonetheless, at the level of potential beneficiaries of AI predictions, that is, patients, beyond emphasizing accuracy, we contend that they should be allowed to decide if and how advanced AI should be integrated in their care. Suppose the accuracy of the machine is the baseline upon which its permissibility is judged, and trust in its accuracy is the cornerstone of healthcare decisions. In that case, medicine has perhaps not moved away from paternalism. The decision maker has simply evolved from man to machine, irrespective of the logic used for such decisions. Involving patients in the decision on whether to use the AI, and subsequently the AI output, creates a shared responsibility. That is, a patient choosing to use AI in their care accepts the shortfalls in its functioning and chooses to bear some responsibility should the output result in a negative outcome. Furthermore, the patient choosing AI in their care, knowing that only limited opaque knowledge is available, essentially consent to uninformed consent, allowing the informed-decision-making gap to perhaps not be bridged but overlooked at their *own* discretion.

Furthermore, neglecting to consider patients as a credible source of knowledge regarding their own health, with input that ought to be considered when health decisions are being made, is a form of testimonial injustice. Neglecting to build training data sets with the input of the subjects themselves and the evidence-based science also surmounts to testimonial injustice. Failing to empower patients’ autonomy through adequate knowledge sharing by using Black Box AI for patient care is a further personal injustice that shows a lack of respect for the individual.

Equally, education on AI and its drawbacks, specifically about the Black Box, would empower patients to be more conscientious in their decisions. The concern remains that patients may not be aware of the right questions to ask due to the media-hyped drive surrounding AI that lacks substance, and hence, would be satisfied with a machine that can provide no answer on how it works. Patients may be confident if it meets their needs, even if it goes against their values. Empowering patients with knowledge of AI will allow them to appreciate the knowledge-exchange gap that comes with AI use and its implications thereof, allowing them to make an informed decision about its use, if not an informed decision on the process that it used.

At the level of training data, creating training data sets that combine the knowledge of the scientists, the clinicians and the patients as all valuable data sources must involve a level of open-mindedness that embraces the knowledge that science needs to be complemented by human judgement and rationale. For example, chemotherapy is a gold standard for cancer, but is chemotherapy the right treatment for this person with cancer given his psycho, social, religious and moral standing?

This feeds into the gaps that AI creates in light of the Black Box problem – knowledge exchange, responsibility, and informed decision-making gaps. The lack of transparency of creators in what training data is used, the inability of the creators to explain how the Black Box works and the inability of the clinician to explain why the AI output suggested a certain intervention/diagnosis/prognosis creates a three-fold gap in knowledge. The lack of higher awareness in a machine to take responsibility for “wrong” decisions, concurrent with the creators’ and clinicians’ lack of insight into how the decisions were made, creates a gap in who ultimately takes responsibility. The unavailability of information makes the informed consent process negligible, and what is not known cannot be shared. These gaps create a barrier to fostering a patient’s rights through inclusivity, empowerment of autonomy and respect for persons and their values. However, as highlighted above, overcoming these gaps will require extensive reworking of the existing system frameworks, and this can only be achieved through practising open-mindedness that acknowledges science ought to be complemented by human interface and that human interface should not be limited to just scientists and software engineers.

The reader would also be correct to observe that AI is not necessarily doomed because of its Black Box problem. There are multiple uses of AI in healthcare that do not directly affect patient care and may create efficiency in healthcare settings that ultimately improve patient care. Concretely, AI, particularly advanced ones like Deep Learning, could be used to plan clinical rotations or triage emergency responses at the hospital and patient administration levels. Who ought to have charge of what aspects of a patient who is coding?

Overall, patient administration is another area in which AI may be used to improve current structures. That is file management, organizing and storing digital records, and retrieving historical information. These are labour and time-intensive jobs that historically would have required many additional hands. However, AI is a suitable alternative to manage these tasks efficiently, and may have the added advantage of minimizing human error and creating easy accessibility routes to retrieving historical data.

An equally potential area that AI may be useful in is patient triage, particularly in high patient influx healthcare settings. Effective triage by AI will assimilate the seriousness and line patients in order of priority. It will also learn to alert emergency services of hospital capacity and redirect ambulances to other centres as needed. Staff responsible for these mundane tasks can then be reallocated to the floor, allowing for quicker patient assessment and management turnover over time.

### Will an interpretable/explainable AI solve the problem definitively?

This section and the subsequent ones address potential criticisms. Notably, a critic may argue that if Deep Learning opacity could be overcome, then the Black Problem would no longer exist. This will undermine the quality of this manuscript’s argument. Precisely, the position this project defends is only relevant because these advanced AI types are opaque. This is the most pressing challenge for integrating this technology into patient care. However, if the processes of the AI were visible and could be understood, then data representation and effective use of all influencing factors, including socioeconomic, religious, cultural and value systems, could be confirmed. Furthermore, users would be able to provide information to patients for comprehensive informed consent. We would be able to study how AI promotes social relations or fosters epistemic justice.

There are many ways to address this criticism. First, interpretable AI research is currently unproductive. Perhaps such studies may yield success in the future, implying that it could give engineers and users greater capacity to understand how an advanced AI works to yield or rank different recommendations. In that case, we may be able to study how AI fosters epistemic justice or promotes individual social relations. However, the critic would notice that an AI may still fail to promote social relations or foster epistemic justice even if it is interpretable. There may be existing and un/recognized biases that the AI may pick from the training data or domain of operation that could still cause the AI to function in problematic ways. If users and developers are not vigilant, these biases may cause injustice to the patients. What this project’s argument points to is that several factors must be present to align advanced AI and its use with key values of social relations and epistemic justice beyond interpretability or explainability. For example, our social contexts or environment must also be free of biases that cause harm to others.

Second, this criticism faces another important problem since it does not align broadly with research on AI. Many authors are convinced that AI opacity will only increase with further advancement in the field. For example, David Beer (2023) has remarked that “the story of neural networks tells us that we are likely to get further away from that objective [of AI explainability/interpretability] in the future, rather than closer to it”. That is, an inverse relationship exists between the two. *The greater the impact AI comes to have on people’s lives, the less they will understand why and how*. Unlike White Box AI and Grey Box AI, Black Box AIs have higher predictive power mostly because of their inherent opacity. Summarily, *advancing* AI technology does not solve the problem of unexplainability (or uninterpretability), but only worsens the same. It seems explainability and interpretability can *effectively* be accomplished by giving AI less predictive power. However, many AI engineers will be unwilling to follow this path since it would amount to retrogression.

Finally, even if interpretable AI had to surpass all these barriers and the vestiges of unethical biases were eradicated from the training data or domain of operation, AI would still be subject to complex proprietary patents, and the transparency may be limited to the creators of the AI. This would result in the clinicians and patients being left in a metaphorical Black Box, in which they are still blind to the processes of the machine.

### Should accuracy be prioritized over the patient’s values or knowledge?

Another objection that may be raised against our argument is that this manuscript’s thesis unjustifiably dismisses the science of AI backed by concrete scientific research with validated results. AI in patient care has been designed after consultation with experts who have contributed extensive knowledge on the subject matter to ascertain safe and accurate functionality. It (AI) is a product of extensive research. In light of the preceding points, what matters is that an AI can enhance patient care, not whether we can or ought to understand how it does this.

In response, the reader would be correct to observe that the accuracy of the machine was never in question. However, it is far more important that the machine can function ethically. Notably, it would be important to understand how an AI promotes a patient’s values to transition patient care in the era of AI to a patient-centred model. In the Jehovah’s Witness example where a blood transfusion is being recommended, it is scientifically accurate that a patient with extremely low haemoglobin will benefit from a blood transfusion, and scientifically that blood transfusion if matched correctly, is unlikely to cause any physical harm. However, that does not account for the patient’s religious standing on the matter or the psycho-social harm and stigmatization that may result from that blood transfusion. It does not account for whether knowledge from a Jehovah’s Witness was used in the creation of training data sets, or if it was included, whether the opaque system considered it when producing the output. It does not provide sufficient information explaining why that is the recommended option when attaining informed consent for that transfusion to occur. It also does not account for what a proxy decision maker should do if they are forced into a position where they are required to make this decision for a Jehovah’s Witness, even though it may not reflect their view or that of the healthcare establishment. In the absence of these explanations, AI-use risks technologizing paternalism in patient care.

### Is human oversight in AI use always possible?

A critic may object that AI use in patient care *will not* exclude human oversight and/or interference. They may point out the numerous propositions to regulate its use in this regard, such as the 2021 proposal for the European Union regulatory framework on artificial intelligence. They may further point out that even if the machine were to be autonomous, the patient, as the human counterpart, can say “no” if the intervention (for example) does not align with their personal values. The control would ultimately lie with the clinician and/or the patient and, in the case of shared decision-making, with the family.

The question about human oversight/interference – and the extent to which this can be realized – in AI use is important since it impacts accountability and informed consent. Although a full deployment of AI in health care is projected to occur by 2035 (Bohr and Memarzadeh 2020), the reality is that some robots have been successfully deployed to perform delicate tasks without human interference or oversight. In light of this, it is possible that AI is already becoming a substitute for human surgeons. For example, engineers at Johns Hopkins developed a robot that autonomously performed laparoscopic surgery on the soft tissue of a pig (Saeidi et al. 2022). Equally, Neuralink recently secured approval from the US FDA to begin a clinical trial to use a surgical robot that will (*likely* independently) implant Brain-Computer Interface in humans (Neuralink, 2023). This does not imply a complete absence of human oversight in the use of automated machines, however, it demonstrates a gradual shift away from it to enable autonomous systems that can decrease human workload and human error. We also suspect that the black-boxed nature of more advanced AI algorithms, like Deep Learning algorithms, will complicate the requirement of human oversight. Notably, suppose a system is intrinsically opaque currently. In that case, we negate that a human can assess the equity with which the black-boxed AI processes data internally to give outputs that will influence human decisions and the required oversight needed to overcome the shortcomings of the technology in that regard. Without knowledge of an AI’s internal operations, it is (nearly) impossible to determine whether and how a patient’s views were considered *in an AI’s internal processes*, even if such views were included in the training data. This suspicion is further strengthened (as stated earlier) by reports which confirm that attempts to increase AI’s internal interpretability/explainability have been mostly unproductive. Did the AI consider patients’ values, or did the AI ignore them? Although a patient can reject the implementation of certain AI outputs, a patient’s inputs in the generation/development of such outputs are equally important to respect them. A different article has reasonably justified this position (Ewuoso, 2023). Moreover, even if global AI regulations were in place, ensuring human oversight in its use and emphasizing the rights of patients to say “no”, neither of these hold the machine accountable for producing outputs that later cause harm to patients. This reinforces machine paternalism. We are unconvinced that machine paternalism is compatible with *patient-centred care*.

## Does drawing on more than one framework undermine this work?

Another objection that may be raised against our argument is that we have used more than one moral concept to argue our point. One might argue that using two moral concepts – epistemic (in)justice and social identity – undermines our capacity to think critically about the concepts, the contestations behind these concepts and the implications that they may have for the use of AI in patient care in light of the Black Box problem.

This criticism points to the argument that this manuscript has failed to ethically engage the concepts and values it draws on. Ethical engagement with theories and frameworks, as Anye-Nkwethi Nyamnjoh and Cornelius Ewuoso (2023) have argued, ought to be deep engagement that consists of, (i) critically developing the justificatory work these concepts are meant to serve and (ii) engaging with the scholarly debates around these concepts that might, in fact, have implications for the project’s thesis. For example, contestations exist regarding whether promoting social relations (a consequentialist reading of social identity in relational autonomy) is more important than how these social relations are promoted (a deontological reading of social relations). Medical AI Black Box is only problematic for social relations if how these relations are promoted matters for social identity. But, some scholars appear to think that what matters is *that* social relations are promoted and not *how* (Jegede 2009). Suppose a medical Black Box AI can promote social relations. In that case, how it does it is no longer relevant, at least from the consequentialist reading of the social identity in relational autonomy.

In response, although these two moral concepts may seem only loosely related, the moral norms that arise from them do have overarching similarities. Furthermore, this is not problematic as the primary objective of this research is evaluative. Precisely, it aims to defend the usefulness of these moral concepts in relation to the research question. As opposed to if our aim were descriptive, in which case a deeper look into the moral concepts would have been relevant.

Furthermore, medical Black Box AI that promotes social relations is no longer acceptable if that promotion is not fair and inclusive and respects the person and their values. If an AI promotes social relations, but only reflects historical privilege, then yes, it is promoting social relations, but it fails to guarantee any form of equity. As we have highlighted, there are multi-dimensions that need to be addressed for the value of promoting social relations to be fully realized.

Notwithstanding, it is worth stating that the requirement to engage concepts deeply is sound. However, does our decision to draw on two values undermine the project’s thesis in any way? No, it does not. Suppose that the ethical concepts had to be engaged more deeply, and a full descriptive component of this paper had been included; it would not change the argument or the outcome. Deep thinking of these ethical concepts had to have occurred in order to achieve the evaluative goal of this thesis. Reflectively, the arguments made would remain, irrespective of the depth.

## Conclusion

The integration of AI in healthcare is an ongoing process, and within the next 5–10 years, it is likely to reach its full potential. As promising as the prospect remains, many challenges with the use of AI in healthcare exist, particularly in light of the Black Box problem. Drawing on the moral norm arising from epistemic (in)justice and social identity in the works of relational autonomists, we have shown how using AI in patient care is problematic in light of its Black Box problem. However, its use cannot be dismissed entirely, as it can be a tool used in non-patient-centred tasks to improve efficiency and functionality in the healthcare setting.

Although we outlined many recommendations in the previous sections, it is worth stating that to overcome the gaps presented by Black Box AI, a level of open-mindedness needs to be adopted in the AI development process to include various underrepresented groups as valuable sources of knowledge, to train underrepresented groups with the necessary skills to be part of the developing teams of AI, and to empower patients with the knowledge they would need to make decisions about themselves with the rising use of AI. Deeper thinking about the impact of AI on the human experience needs to be done to appreciate any other barriers that may exist from other backgrounds other than decolonial scholarship and relational autonomy. Future studies can take up this task.

## Data Availability

No new data were created or analysed in this study. Data sharing is not applicable to this article.
